# Genetic and isotope ratio mass spectrometric evidence for the occurrence of starch degradation and cycling in illuminated Arabidopsis leaves

**DOI:** 10.1371/journal.pone.0171245

**Published:** 2017-02-02

**Authors:** Marouane Baslam, Edurne Baroja-Fernández, Adriana Ricarte-Bermejo, Ángela María Sánchez-López, Iker Aranjuelo, Abdellatif Bahaji, Francisco José Muñoz, Goizeder Almagro, Pablo Pujol, Regina Galarza, Pilar Teixidor, Javier Pozueta-Romero

**Affiliations:** 1 Instituto de Agrobiotecnología (CSIC/UPNA/Gobierno de Navarra). Iruñako etorbidea 123, Mutiloabeti, Nafarroa, Spain; 2 Servicio de Apoyo a la Investigación, Universidad Pública de Navarra, Campus de Arrosadia, Iruña, Nafarroa, Spain; 3 Centres Científics i Tecnològics, Universitat de Barcelona, C/ Lluís Solé I Sabarís 1–3, Barcelona, Spain; RIKEN Center for Sustainable Resource Science, JAPAN

## Abstract

Although there is a great wealth of data supporting the occurrence of simultaneous synthesis and breakdown of storage carbohydrate in many organisms, previous ^13^CO_2_ pulse-chase based studies indicated that starch degradation does not operate in illuminated Arabidopsis leaves. Here we show that leaves of *gwd*, *sex4*, *bam4*, *bam1/bam3* and *amy3/isa3/lda* starch breakdown mutants accumulate higher levels of starch than wild type (WT) leaves when cultured under continuous light (CL) conditions. We also show that leaves of CL grown *dpe1* plants impaired in the plastidic disproportionating enzyme accumulate higher levels of maltotriose than WT leaves, the overall data providing evidence for the occurrence of extensive starch degradation in illuminated leaves. Moreover, we show that leaves of CL grown *mex1/pglct* plants impaired in the chloroplastic maltose and glucose transporters display a severe dwarf phenotype and accumulate high levels of maltose, strongly indicating that the MEX1 and pGlcT transporters are involved in the export of starch breakdown products to the cytosol to support growth during illumination. To investigate whether starch breakdown products can be recycled back to starch during illumination through a mechanism involving ADP-glucose pyrophosphorylase (AGP) we conducted kinetic analyses of the stable isotope carbon composition (δ^13^C) in starch of leaves of ^13^CO_2_ pulsed-chased WT and AGP lacking *aps1* plants. Notably, the rate of increase of δ^13^C in starch of *aps1* leaves during the pulse was exceedingly higher than that of WT leaves. Furthermore, δ^13^C decline in starch of *aps1* leaves during the chase was much faster than that of WT leaves, which provides strong evidence for the occurrence of AGP-mediated cycling of starch breakdown products in illuminated Arabidopsis leaves.

## Introduction

A substrate or “futile” cycle is a metabolic cycle of simultaneous synthesis and breakdown of a compound for which the net balance consists solely on the dissipation of energy [1,2]. In carbon cycles, energy dissipation occurs mainly, but not exclusively, through the net hydrolysis of ATP. In some cases substrate cycles consume up to 70% of the ATP produced by the cell [3–6]. They operate in microorganisms [2], plants [7,8], yeasts [9] and animals [10], playing roles such as heat generation, buffering of metabolite concentrations, improvement of sensitivity in metabolic regulation, and control of the direction of flow in bidirectional pathways [1,2]. In particular, there is a great wealth of genetic, radiotracer, stoichiometric analysis and stable isotope labeling data supporting the occurrence of metabolic cycles resulting from the simultaneous synthesis and breakdown of storage carbohydrates such as trehalose in fungi [11,12], sucrose and starch in heterotrophic organs of plants [7,13,14] and glycogen in animals [15–17], yeasts [11] and bacteria [18–24].

Starch is the main storage carbohydrate in plants. Synthesized by starch synthases (SS) using ADP-glucose (ADPG) as the sugar donor molecule, and branching enzymes, this polyglucan accumulates in photosynthetic and non-photosynthetic tissues of plants. In mesophyll cells of leaves, up to 50% of the photosynthate is retained within the chloroplasts during the day in the form of starch [25], which is then remobilized during the night to support nonphotosynthetic metabolism and growth. Starch is made of two distinct polysaccharide fractions that are assembled together to form a semi-crystalline starch granule: amylose and amylopectin. Amylose is a linear polymer of up to several thousand glucose residues, whereas amylopectin is a larger polymer regularly branched with α-1,6-branch points exhibiting hierarchical levels of specific architectural structure [26,27] whose synthesis requires the highly coordinated actions of SSs, branching and debranching enzymes [28].

It is widely accepted that, in mesophyll cells of illuminated leaves, the whole starch biosynthetic process resides exclusively in the chloroplast [28]. According to this view, starch is considered the end-product of a unidirectional pathway that is linked to the Calvin-Benson cycle (CBC) by means of the plastidic phosphoglucose isomerase (pPGI) (**S1A Fig**). This enzyme catalyzes the conversion of fructose-6-phosphate (F6P) from the CBC into glucose-6-phosphate (G6P), which is then converted into ADPG linked to starch biosynthesis by the stepwise reactions of plastidic phosphoglucomutase (pPGM) and ADPG pyrophosphorylase (AGP). This interpretation of starch biosynthesis implies that AGP is the sole source of ADPG, and functions as the major regulatory step in the starch biosynthetic process [29,30]. In *Arabidopsis*, genetic evidence showing that transitory starch biosynthesis occurs solely by the CBC-pPGI-pPGM-AGP-SS pathway has been obtained from the characterization of mutants impaired in pPGI, pPGM and AGP. Leaves of mutants totally lacking either pPGM and/or AGP accumulate ca. 1–3% of the wild type (WT) starch, whereas leaves impaired in pPGI accumulate ca. 10% of the WT starch [31–39].

The pathway of nocturnal starch breakdown in mesophyll cells of leaves is relatively complex and requires the coordinated actions of a suit of enzymes (**S1B Fig**) [28]. The initial steps involve the phosphorylation of the starch granule surface by enzymes of the glucan, water dikinase class (GWD) and phosphoglucan, water dikinase [28]. Removal of the phosphate groups by the phosphoglucan phosphatase SEX4 is also required for proper starch metabolism [28]. A set of enzymes then degrades starch via a network of reactions to maltose, glucose and G1P, the former two sugars being exported to the cytosol via the MEX1 and pGlcT transporters, respectively, to be subsequently converted into sucrose [28,40]. Maltose can be produced by chloroplastic β-amylases 1–3 (BAM1-3), which act at the starch granule surface or on malto-oligosaccharides produced by α-amylase 3 (AMY3), and the ISA3 and LDA debranching enzymes [28]. BAM4, a noncatalytic protein, is also required for starch breakdown and acts upstream of BAM1-3 [28]. Glucose can be produced from malto-oligosaccharides by the disproportionating enzyme 1 (DPE1), which transfers a maltosyl unit from the non-reducing end of maltotriose, to another acceptor α-1,4-glucan chain [28]. G1P can be produced by starch phosphorylase (SP), which catalyzes the phosphorolysis of the terminal residue from the nonreducing ends of α-1,4-linked glucan chains of starch [28,41]. In Arabidopsis, genetic evidence demonstrating the predominance of the amylolytic starch breakdown pathway has been obtained from the characterization of mutants such as *gwd*, *sex4*, *bam3*, *bam1/bam3*, *bam4*, *mex1*, *mex1/pglct-1*, *dpe1* and *amy3/isa3/lda*. Leaves of these plants cultured under short day (SD) (12 h light/12 h dark) conditions accumulate high levels of starch, a phenotype that has been ascribed to reduced starch degradation during the dark period [28].

Evidence has been provided that, in addition to the CBC-pPGI-pPGM-AGP-SS pathway, plants possess additional/alternative pathway(s) of transitory starch biosynthesis wherein (i) hexose-phosphates and/or ADPG occurring in the cytosol enter the chloroplast for subsequent conversion into starch, (ii) CBC and the pPGM-AGP-SS starch biosynthetic pathway are not connected by pPGI, and (iii) starch synthesis and breakdown simultaneously occur during illumination thus allowing the formation of a substrate (starch) cycle wherein pPGM and AGP (but not pPGI) play an important role in recycling back to starch its own breakdown products [32,38,39,42,43]. According to this interpretation, starch accumulation in illuminated leaves would be the result of the balance between starch synthesis and degradation, and the efficiency by which starch breakdown products are recycled back to starch.

Supporting the occurrence of starch breakdown during illumination in leaves, several independent studies have shown that most of the enzymes involved in starch breakdown are redox-activated at physiologically relevant potentials occurring in the illuminated chloroplast [44–48]. Also, Caspar et al. [49] reported that *gwd* leaves accumulate higher levels of starch than WT leaves when plants are cultured under continuous light (CL) conditions. Moreover, recent metabolic flux analyses carried out using illuminated Arabidopsis plants cultured in ^13^CO_2_-enriched environment revealed labeling of the starch breakdown product maltose [50]. Additional evidence comes from ^14^CO_2_ pulse-chase and starch-preloading experiments using isolated chloroplasts [51] or cultured photosynthetic cells [52] in which chloroplasts were capable of synthesizing and mobilizing starch simultaneously during illumination. These observations, however, are in apparent conflict with ^14^CO_2_ pulse-chase experiments on illuminated leaves showing no significant loss of ^14^C from starch during the chase period [53,54], which are considered as the cornerstone evidence that starch degradation does not occur during illumination in leaves [28,50,53–55]. Consequently, the widely accepted paradigm on transitory starch metabolism in leaves assumes that starch cycling does not operate since starch breakdown solely occurs during the night under normal growth conditions.

The ^14^CO_2_ pulse-chase method employed to investigate the possible occurrence of starch breakdown in illuminated leaves is based on the enzymatic digestion of ethanol precipitates from crude extracts using amylolytic enzymes (e.g. amyloglucosidase and α-amylase) and measurement of the label in the supernatant [53,54]. However, this method is relatively coarse, does not have the necessary precision to detect rapid turnover such as the debranching steps integral to the maturation of amylopectin, and is prone to the problem that amyloglucosidase and α-amylase hydrolysates from crude extracts can harbor contaminants from non-starch compounds [56]. To investigate whether starch degradation takes place during illumination in Arabidopsis leaves in this work we measured the starch content in different starch breakdown mutants cultured under CL conditions. Furthermore, to investigate the possible occurrence of AGP-mediated cycling of starch breakdown products, we also performed ^13^CO_2_ pulse-chase experiments using WT and AGP null *aps1* plants, and analyzed changes in stable carbon isotope ratios (δ^13^C) in starch by using high-performance liquid chromatography (HPLC) combined with isotope ratio mass spectrometry (IRMS). Contrary to the widely accepted paradigm that starch breakdown solely occurs during the night in Arabidopsis leaves, results presented in this work provide strong evidence supporting the occurrence of extensive starch degradation, and AGP-mediated cycling of starch breakdown products in illuminated Arabidopsis leaves.

## Results and discussion

### Leaves of different starch breakdown mutants accumulate higher levels of starch than WT leaves when cultured under continuous light conditions

**Fig 1:**
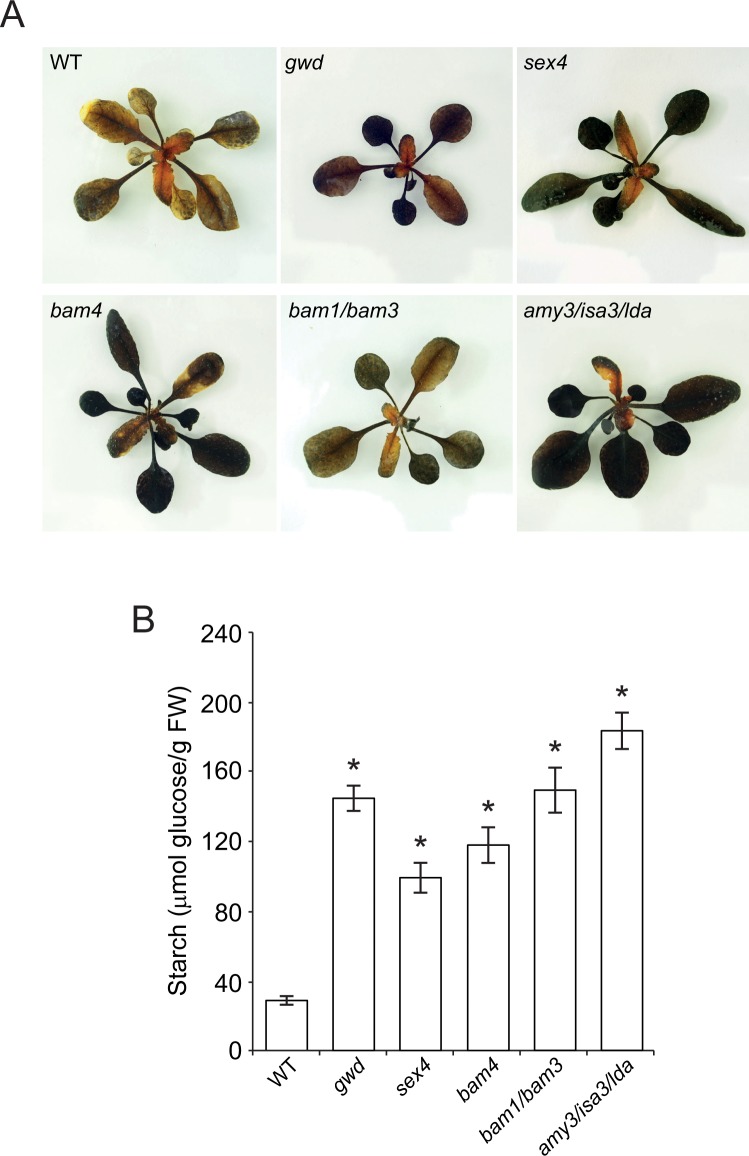
Leaves of different starch breakdown mutants display a high starch content phenotype when cultured under continuous light conditions. (A) Iodine staining and (B) starch content in leaves of WT and the indicated starch breakdown mutants cultured under CL conditions. Leaves were harvested at the 18 days after sowing (DAS) growth stage. In “B” values represent the means ± SE determined from three independent experiments using 6 plants in each experiment. Asterisks indicate significant differences with respect to WT plants according to Student´s t-tests (*p*<0.05).

Caspar et al. [49] reported that *gwd* leaves accumulate higher levels of starch than WT leaves when plants were cultured under CL conditions. This phenomenon can be ascribed to either one of the following reasons: (i) starch degradation operates in WT illuminated leaves (but not in *gwd* leaves), or (ii) starch phosphorylation by GWD exerts a negative effect on starch biosynthesis during illumination. To differentiate between these possibilities we measured the starch content in leaves of *gwd*, *sex4*, *bam1/bam3*, *bam4* and *amy3/isa3/lda* starch breakdown mutants germinated and permanently cultured under CL conditions. The rationale behind this experimental approach was that, if starch breakdown solely occurs during the dark period, leaves of starch breakdown mutants other than *gwd* should accumulate WT levels of starch when cultured in the absence of a dark period. Conversely, if starch degradation also occurs during the day, leaves of the above starch breakdown mutants should accumulate higher levels of starch than WT leaves. As shown in **Fig 1**, both iodine staining and quantitative starch content measurement analyses revealed that leaves of all the above starch breakdown mutants accumulate higher levels of starch than those of WT plants cultured under CL conditions. The overall data thus provide strong evidence for the occurrence of starch breakdown in Arabidopsis leaves during illumination.

To examine for the possible occurrence of pleiotropic effects that could explain the high starch content phenotype in all the above starch breakdown mutants we measured their net photosynthetic CO_2_ fixation rates (*A*_*n*_). As shown in **S2A Fig**, *A*_*n*_ values of the above mutants were comparable to those of WT plants. We also measured the maximum catalytic activities of AGP and carried out non-reducing western blot analyses of the small, catalytically active AGP subunit (APS1), which in leaves is present as a mixture of ca. 50 kDa active (reduced) monomers and ca. 100 kDa inactive (oxidized) dimers formed by intermolecular links involving Cys bridges [57]. These analyses revealed no changes in total AGP activity (**S2B Fig**) and in the levels of ca. 50 kDa APS1 monomers in leaves of WT and starch breakdown mutants (**S2C Fig**). We also measured the maximum catalytic activities of SS and carried western blot analyses of SS4, an important determinant of starch content [58,59]. No differences in total SS activity (**S2B Fig**) and in the levels of SS4 could be found between WT and starch breakdown mutants (**S2D Fig**). Therefore, high levels of starch in leaves of CL grown starch breakdown mutants cannot be ascribed to enhanced photosynthesis or to high SS and/or AGP activities, but more logically to reduced starch breakdown.

### *mex1* and *mex1/pglct* mutants accumulate high levels of maltose and display a dwarf phenotype when plants are cultured under continuous light conditions

**Fig 2:**
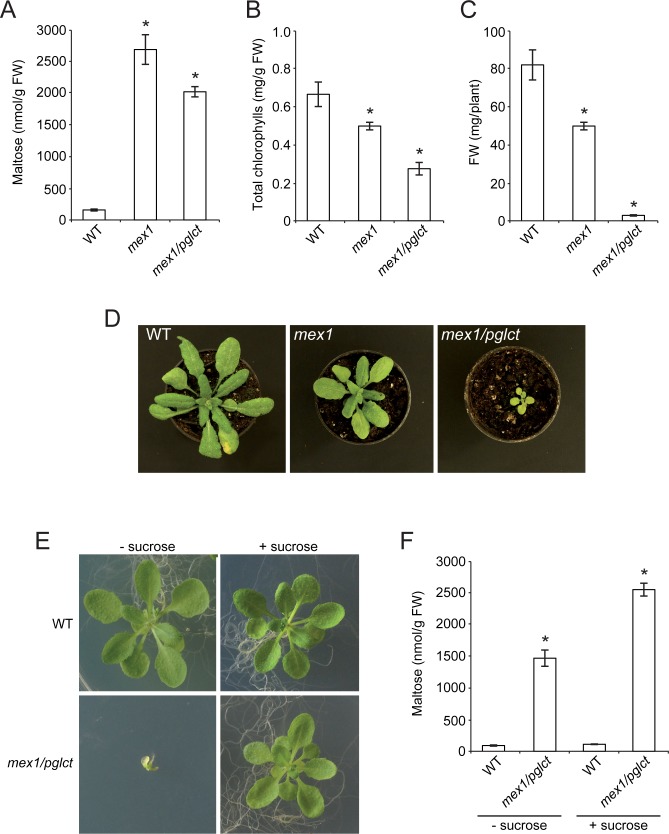
*mex1* and *mex1/pglct* leaves accumulate higher levels of maltose than WT leaves when plants are cultured under continuous light conditions. (A) Maltose content in leaves, (B) total chlorophyll content in leaves, (C) rosette FW and (D) external phenotype of 20 DAS WT (Col-*0*), *mex1* and *mex1/pglct* plants cultured on soil under CL conditions. (E) External phenotype and (F) leaf maltose content in 20 DAS WT and *mex1/pglct* plants cultured on solid MS medium with or without 90 mM sucrose supplementation. In “A”, “B”, “C” and “F” values represent the means ± SE determined from three independent experiments using 6 plants in each experiment. Asterisks indicate significant differences with respect to WT plants according to Student´s t-tests (*p*<0.05).

Maltose is the major starch breakdown product exported from the chloroplast to the cytosol at night as strongly supported by the fact that leaves of the *mex1* mutant impaired in the chloroplastic MEX1 maltose transporter accumulate high levels of maltose when cultured under SD conditions [60]. Lack of maltose transport in *mex1* plants causes growth retardation since nonphotosynthetic metabolism is prevented during the night and maltose over-accumulation causes chloroplast dysfunction [28,60]. This phenotype is even more severe in *mex1/pglct* double mutants impaired in both the MEX1 and the chloroplastic pGlcT glucose transporter [40], indicating that glucose is also an important form of carbon exported to the cytosol at night. To further investigate the possible occurrence of starch breakdown in illuminated leaves we measured the maltose contents in leaves of *mex1* and *mex1/pglct* plants germinated and permanently cultured under CL conditions. We also measured the fresh weight (FW) and chlorophyll contents of these plants. Assuming that amylolytic starch breakdown is the sole source of maltose in Arabidopsis leaves, we reasoned that if starch breakdown solely occurs during the dark period, leaves of WT, *mex1* and *mex1/pglct* plants cultured under CL conditions should not accumulate any maltose. Furthermore, *mex1* and *mex1/pglct* plants should accumulate WT levels of chlorophyll and display WT growth phenotypes. Conversely, if starch degradation also occurs during the day, leaves of WT plants should accumulate maltose when cultured under CL conditions. Furthermore, maltose content in *mex1* and *mex1/pglct* leaves should be higher than that of WT leaves. Moreover *mex1* plants should have reduced chlorophyll content and growth. As shown in **Fig 2A**, leaves of CL grown WT plants accumulated maltose. Notably, levels of this disaccharide in leaves of CL grown *mex1* and *mex1/pglct* plants were exceedingly higher than those of WT leaves (**Fig 2A**). Furthermore, *mex1* plants accumulated low levels of chlorophyll in their leaves (**Fig 2B**), showed reduced photosynthetic capacities (**S2A Fig**) and were small (**Fig 2C and 2D**) when compared with WT leaves. These phenotypes were even more severe in the *mex1/pglct* mutant (**Fig 2B, 2C and 2D**).

To examine for the possible occurrence of pleiotropic effects that could explain the severe dwarf phenotype of the CL grown *mex1/pglct* plants we cultured mutant plants on MS medium with or without sucrose supplementation. As shown in **Fig 2E**, the defective growth of *mex1/pglct* plants was found to be mostly rescued by the external supply of sucrose. It should be emphasized that, irrespective of the presence of sucrose in the culture medium, leaves of CL grown *mex1/pglct* plants accumulated high levels of maltose (**Fig 2F**). Thus, the defective growth of the *mex1/pglct* mutant cultured in sucrose-free medium is neither ascribed to pleiotropic effects nor to maltose over-accumulation, but essentially caused by a shortage of carbohydrates as a consequence of limited photosynthesis and restricted export of photoassimilates from the chloroplast to the cytosol. The overall data thus provide further evidence for the occurrence of starch breakdown in leaves during illumination.

### *dpe1* leaves accumulate higher levels of maltotriose than WT leaves when plants are cultured under continuous light conditions

DPE1 plays an important role in starch degradation as strongly supported by the fact that leaves of the *dpe1* mutant accumulate high levels of maltotriose (the by-product of β-amylolysis of malto-oligosaccharides derived from starch breakdown) when plants are cultured under SD conditions [28]. To further investigate the possible occurrence of starch degradation in illuminated leaves we measured the maltotriose content in leaves of *dpe1* plants germinated and permanently cultured under CL conditions. The rationale behind this experimental approach was that if starch degradation occurs during the day, *dpe1* leaves should accumulate higher levels of maltotriose than WT leaves. In support of this presumption, we found that maltotriose levels in leaves of *dpe1* plants cultured under CL conditions (120.2 ± 17.1 nmol/g FW) were exceedingly higher than those of WT leaves (9.3 ± 1.1 nmol/g FW).

### Kinetic analyses of δ^13^C in starch of WT and *aps1* plants exposed to ^13^C enriched CO_2_ provide evidence for the occurrence of starch cycling through an AGP-mediated mechanism

The chloroplast is equipped with a set of enzymes (i.e. plastidic hexokinase, pPGM, SP and AGP) that are capable of recycling the starch breakdown products into starch [42]. We next investigated whether starch breakdown products can be recycled back to starch through an AGP mediated process. The classic ^14^CO_2_ pulse-chase method employed to investigate the possible occurrence of starch breakdown and cycling in illuminated leaves is based on the measurement of the ^14^C-label in amyloglucosidase and α-amylase digests of ethanol precipitates obtained from crude extracts of plants [53,54]. However, this method is prone to the problem that the enzymatic digests can exhibit contaminations by non-starch compounds [56].

**Fig 3:**
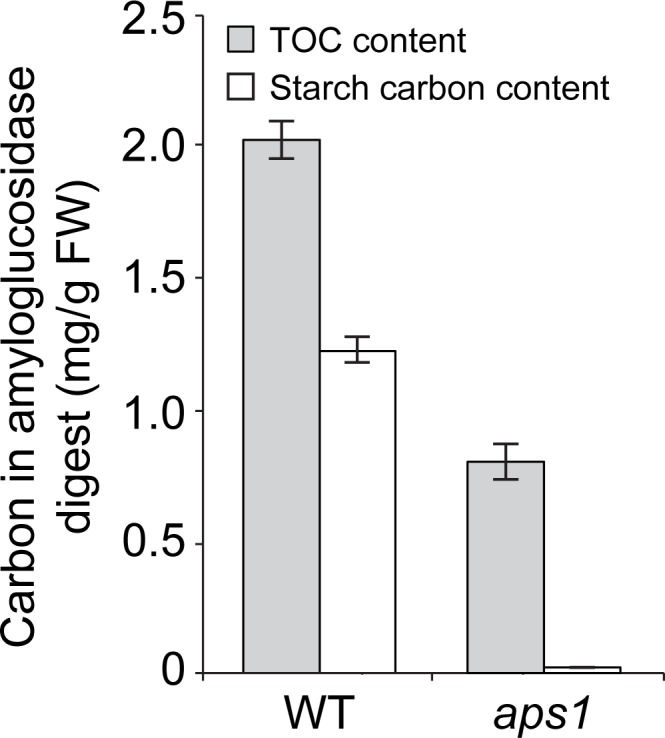
Amyloglucosidase releases carbon compounds other than starch glucose molecules from Arabidopsis leaf ethanol precipitates. The graphic shows the total carbon (TOC) and the starch carbon content in amyloglucosidase digests of WT (Col-*0*) and *aps1* leaves. Values represent the means ± SE determined from three independent experiments using 6 plants in each experiment.

As a first step to investigate the possible occurrence of AGP-mediated mechanisms of starch cycling in illuminated Arabidopsis leaves we explored whether amyloglucosidase digestion of Arabidopsis leaf ethanol precipitates is capable of releasing carbon compounds other than starch-based glucose molecules. Towards this end, we compared the starch carbon and total carbon (TOC) contents in amyloglucosidase digests of leaves of WT and *aps1* plants. As shown in **Fig 3**, these analyses revealed that TOC contents are higher than starch carbon contents in both WT and *aps1* amyloglucosidase digests, which shows that amyloglucosidase is capable of releasing carbon compounds other than starch glucose molecules from Arabidopsis leaf ethanol precipitates. Therefore, starch cycling studies in illuminated leaves should be based on methods capable of discriminating between carbon from starch compounds (glucose) and non-starch compounds in amyloglucosidase digests.

Because of its precision, sensitivity and accuracy, HPLC/IRMS is now considered the most suitable technique for measuring stable carbon isotope (^13^C/^12^C) ratios, isotopic enrichments and turnover of a variety of carbohydrates [61,62]. Thus, to explore the possible occurrence of AGP-mediated recycling of starch breakdown products we performed ^13^CO_2_ pulse-chase experiments using WT and *aps1* plants cultured under long day (LD) conditions in ^13^CO_2_-enriched environment, and carried out time-course HPLC/IRMS analyses of δ^13^C in the glucose molecules of amyloglucosidase digests obtained as illustrated in **S3 Fig**. We also used the very low starch *pgi1-2* mutant as control. We reasoned that if AGP-mediated starch cycling occurs during illumination, the rate of increase of δ^13^C in starch of *aps1* leaves during the pulse should be higher than that of WT leaves and *pgi1-2* leaves, since only glucose molecules coming from newly fixed ^13^C enriched CO_2_ will be incorporated into starch in *aps1* leaves. In contrast, glucose molecules derived from both newly fixed ^13^C enriched CO_2_ and ^13^C non-enriched starch breakdown products will be incorporated into starch in WT and *pgi1-2* leaves. Moreover, reduction of δ^13^C in starch of *aps1* leaves during the chase should be faster than that of WT and *pgi1-2* leaves. This would be the case since glucose molecules derived from the breakdown of ^13^C enriched starch will not be recycled back to starch in *aps1* leaves, and only glucose molecules coming from newly fixed ^13^C non-enriched CO_2_ will be incorporated into starch. In contrast, glucose molecules from both newly fixed ^13^C non-enriched CO_2_ and ^13^C enriched starch breakdown products will be incorporated into starch in WT and *pgi1-2* leaves. Conversely, if starch cycling does not operate during illumination, δ^13^C kinetics in starch during the ^13^CO_2_ pulse and chase in *aps1* leaves should be comparable to that of WT and *pgi1-2* leaves.

**Fig 4:**
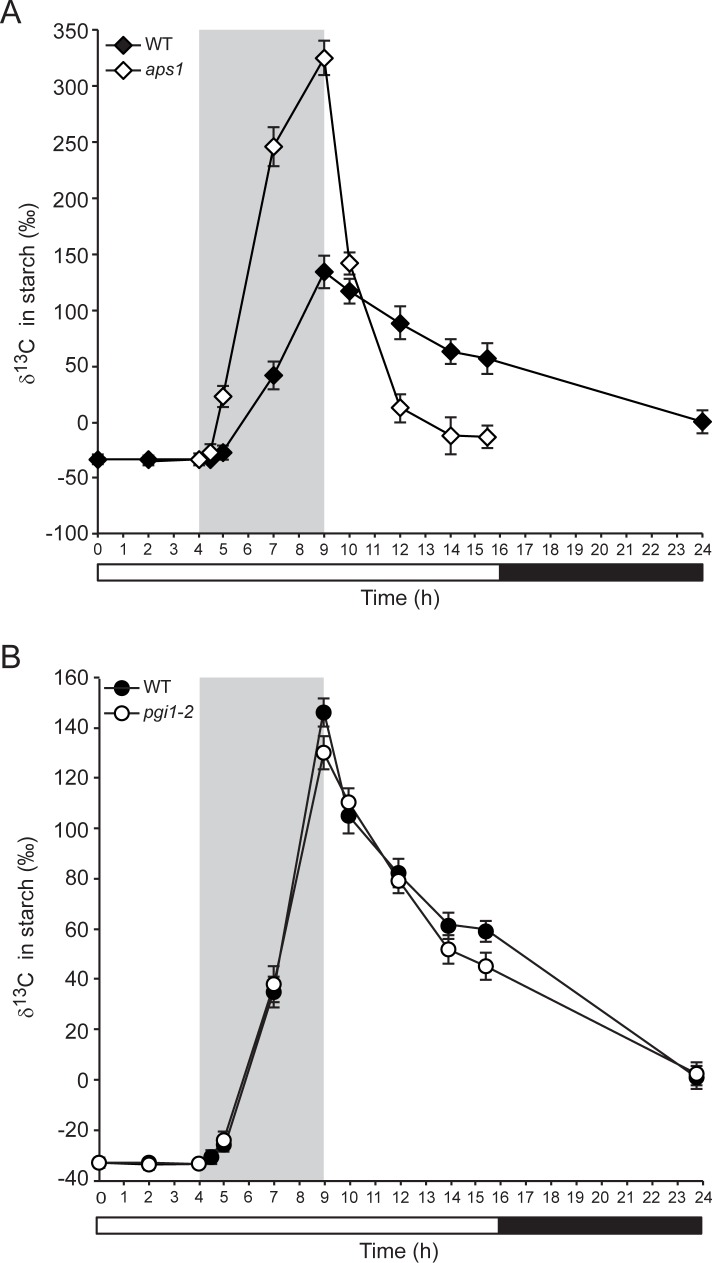
Isotope ratio mass spectrometric evidence for the occurrence of starch cycling in illuminated leaves through a mechanism involving AGP. The graphics represent the values of δ^13^C in starch of leaves of (A) 26 DAS WT (Col-*0*) and *aps1* plants, and (B) 22 DAS WT (Ws-2) and *pgi1-2* plants exposed to ^13^C enriched CO_2_ for 5 hours and then chased for 15 additional hours. Plants were cultured in growth cabinets under long day (LD) conditions. The grey area indicates the ^13^CO_2_ pulse period. Starch content in leaves is shown in **S4 Fig**. Values represent the means ± SE determined from three independent experiments using 6 plants in each experiment.

As shown in **S4 Fig**, time-course analyses of starch content confirmed that *pgi1-2* and *aps1* leaves accumulate very low levels of starch when compared with their respective WT plants (Ws-2 and Col-*0*, respectively). As expected, δ^13^C values in starch of both WT and *aps1* leaves increased during the pulse and then dropped during the subsequent chase (**Fig 4A**). Notably, in strong support of the idea that AGP-mediated starch cycling occurs in illuminated leaves, ^13^C enrichment in starch of *aps1* leaves during the pulse was faster than that of WT leaves (**Fig 4A**). Furthermore δ^13^C decline in starch of *aps1* leaves during the chase was faster than that of WT leaves (**Fig 4A**). This is especially meaningful considering that δ^13^C kinetics in starch of *pgi1-2* leaves during the ^13^CO_2_ pulse and chase was comparable to that of WT leaves (**Fig 4B**), indicating that pPGI is not involved in starch cycling.

A possibility cannot be ruled out that the differences observed in the δ^13^C kinetics in starch of *aps1* and WT plants could be the consequence, at least partly, of the exchange of glucose moieties at the granule surface with free sugars or sugar phosphates by SP or DPE1, which in the very low starch *aps1* mutant may be accentuated since the surface area to volume ratio of granules is very much greater than in WT plants. However, this scenario is highly unlikely since the δ^13^C kinetics in starch of WT and *pgi1-2* leaves were very similar (**Fig 4B**).

### δ^13^C kinetics in starch is slower than that of sucrose in WT plants cultured in ^13^CO_2_-enriched environment

Previous studies of ^13^C enrichment in different metabolites of primary metabolism in Arabidopsis plants cultured in ^13^CO_2_-enriched environment showed that labeling kinetics in CBC intermediates are much faster than that of sucrose [50]. Provided starch is synthesized from CBC intermediates in the chloroplast, while sucrose is synthesized from CBC intermediates that are exported to the cytosol (**S1A Fig**), these results implied that starch would label faster than sucrose. However, kinetic analyses of ^13^C enrichment in starch were not performed [50].

**Fig 5:**
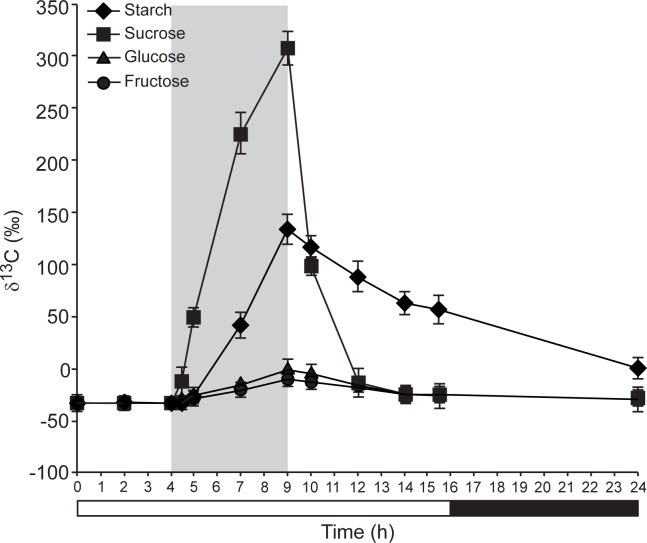
δ^13^C kinetics in starch is slower than that of sucrose in WT plants cultured in ^13^CO_2_-enriched environment. The graphic represents the values of δ^13^C in starch and the indicated soluble sugars of leaves of 26 DAS WT (Col-*0*) plants exposed to ^13^C enriched CO_2_ for 5 hours and then chased for 15 additional hours. Plants were cultured in growth cabinets under long day (LD) conditions. The grey area indicates the ^13^CO_2_ pulse period. Values represent the means ± SE determined from three independent experiments using 6 plants in each experiment.

To further investigate the possible occurrence of starch cycling in illuminated leaves we carried out kinetic analyses of δ^13^C in the sucrose of ^13^CO_2_ pulsed-chased WT leaves and compared them with that of starch. We also analyzed the δ^13^C kinetics of glucose and fructose. We reasoned that if starch cycling does not operate during illumination, δ^13^C kinetics in starch during the ^13^CO_2_ pulse and chase should be faster than that of sucrose, especially under conditions of very active starch accumulation. Conversely, if starch cycling occurs, δ^13^C kinetics in starch during the ^13^CO_2_ pulse and chase should be comparable or even slower than that of sucrose. As shown in **Fig 5**, sucrose labeled faster than glucose and fructose, which is consistent with [50]. Notably, the rate of increase of δ^13^C in sucrose during the pulse was exceedingly higher than that of starch, even under conditions of most active starch accumulation (**Figs 4** and **5, S4 Fig**). Furthermore δ^13^C decline in sucrose during the chase was faster than that of starch (**Figs 4** and **5**). Overall, these data are consistent with the idea that starch is subject to continuous cycling during illumination.

### *pgm* and *aps1* leaves simultaneously synthesize and degrade starch during illumination

The operation of starch cycling through a mechanism involving AGP, and the occurrence of WT levels of ADPG in *aps1*, *pgm* and *aps1/pgm* leaves [38,39] are consistent with the idea that mutants totally lacking pPGM and AGP synthesize starch from ADPG produced by metabolic pathways other than the CBC-pPGI-pPGM-AGP pathway, but starch accumulation is prevented, at least partly, by the blockage of the mechanism of scavenging of starch breakdown products [39].

To investigate the possible occurrence of simultaneous synthesis and breakdown of starch in mutants impaired in pPGM and AGP during illumination we conducted high-performance anion-exchange chromatography with pulsed amperometric detection (HPAEC-PAD) and gas chromatography-mass spectrometry (GC-MS) analyses of the maltose contents in leaves of *pgm*, *aps1*, *aps1/pgm*, *mex1/pgm* and *mex1/aps1* plants cultured under LD and CL conditions, and compared them with that of WT leaves. The rationale behind this experimental approach was that if *pgm*, *aps1* and *aps1/pgm* leaves do not actively synthesize starch, they should not accumulate any maltose. Conversely, if *pgm*, *aps1* and *aps1/pgm* leaves synthesize starch, they should accumulate substantial levels of maltose. Furthermore, *mex1/pgm* and *mex1/aps1* leaves should accumulate higher levels of maltose than *pgm* and *aps1* leaves.

**Fig 6:**
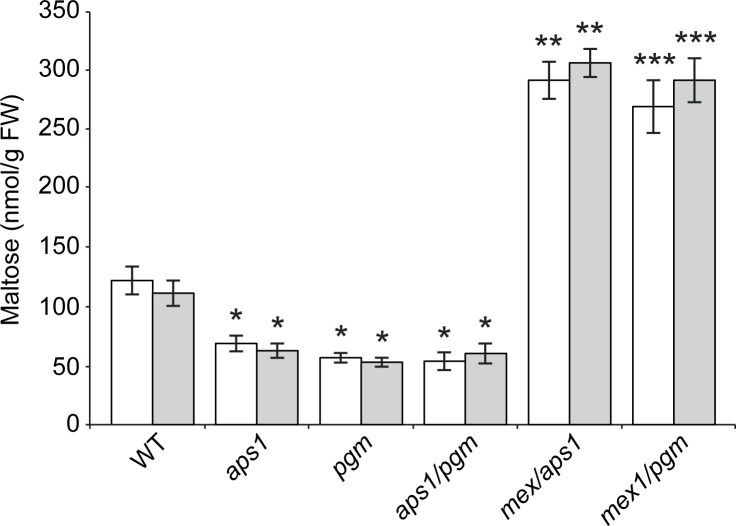
Maltose content in leaves of WT (Col-*0*), *aps1*, *pgm*, *aps1/pgm*, *mex1/aps1* and *mex1/pgm* plants. Leaves of the indicated plants were harvested at the 18 DAS growth stage. Values obtained using HPAEC-PAD and GC-MS are represented with white and grey columns, respectively. Values represent the means ± SE determined from three independent experiments using 6 plants in each experiment. Asterisks indicate significant differences according to Student´s t-tests (*P<0.05, *aps1*, *pgm* and *aps1/pgm* vs. Col-*0*; **P<0.05, *mex1/aps1* vs. *aps1;* ***P<0.05, *mex1/pgm* vs. *pgm*). Values correspond to plants cultured under continuous light (CL) conditions. Essentially the same results were obtained using plants cultured under long day (LD) conditions (not shown).

Previous HPAEC-PAD analyses of maltose content in *pgm* leaves presented contrasting results. Thus, whereas [63] reported that *pgm* leaves accumulate WT levels of maltose during illumination, [64] reported that *pgm* leaves do not accumulate any maltose. Therefore, to correctly identify maltose by HPAEC-PAD we added known amounts of maltose to extracts of leaves that had been previously digested with maltase, an enzyme that catalyzes the hydrolytic breakdown of maltose. Further confirmed by GC-MS, these analyses revealed that leaves of *pgm*, *aps1* and *aps1/pgm* plants accumulate ca. 40–50% of the WT maltose content under both LD and CL conditions (**Fig 6** and data not shown). Moreover, *mex1/aps1* and *mex1/pgm* leaves accumulated exceedingly higher levels of maltose than *aps1* and *pgm* leaves, and ca. 3-fold more maltose than WT leaves in the two photoregimes (**Fig 6** and data not shown).

**Fig 7:**
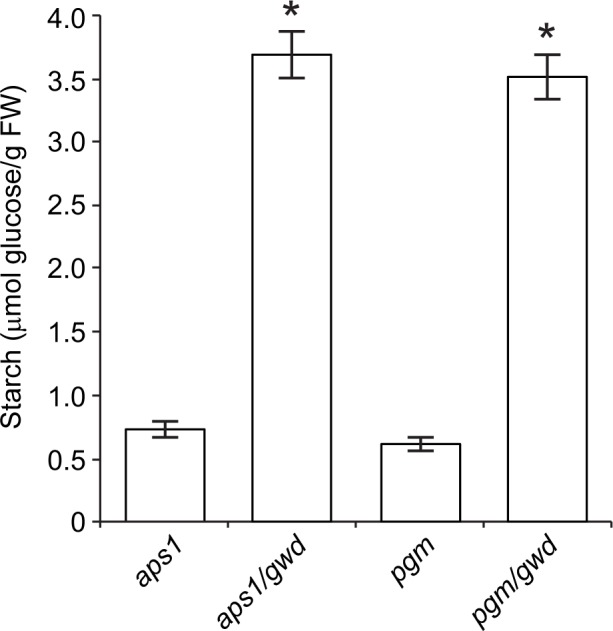
Incorporation of the *gwd* mutation enhances starch content in leaves of continuous light grown *pgm* and *aps1* plants. The graphic represents the starch content in 20 DAS *aps1*, *aps1/gwd*, *pgm* and *pgm/gwd* leaves. Values represent the means ± SE determined from three independent experiments using 6 plants in each experiment. Asterisks in *aps1/gwd* and *pgm/gwd* indicate significant differences with respect to *aps1* and *pgm* plants according to Student´s t-tests (*p*<0.05).

We also measured the starch contents in leaves of CL grown *pgm/gwd* and *aps1/gwd* plants, and compared them with those of *pgm* and *aps1* leaves. We reasoned that if starch breakdown occurs in leaves of CL grown *pgm* and *aps1* plants, *pgm/gwd* and *aps1/gwd* leaves should accumulate higher levels of starch than *pgm* and *aps1* leaves. In line with this presumption, we found that *pgm/gwd* and *aps1/gwd* leaves accumulate ca. 5-fold more starch than *pgm* and *aps1* leaves (**Fig 7**), which corresponds to 10–15% of the WT starch (cf. **Fig 1B**). Similar results were obtained using plants cultured under LD conditions (not shown). To examine for the possible occurrence of pleiotropic effects that could explain the enhanced levels of starch by *gwd* incorporation we measured *A*_*n*_ under varying *Ci* in WT, *gwd*, *aps1* and *aps1/gwd* plants. Consistent with [65,66], we found that *A*_*n*_ values in *aps1* leaves are lower than those of WT at any *Ci* (**S5 Fig**), which could explain to some extent the low starch content of this mutant. Moreover, *A*_*n*_ values in *aps1/gwd* leaves were still lower than those of *aps1* leaves, indicating that incorporation of the *gwd* mutation exerts a negative effect on the photosynthetic capacity of *aps1* plants. Therefore, enhanced levels of starch in leaves of CL grown *aps1/gwd* plants can logically be ascribed to reduced starch breakdown.

The overall data thus provide strong evidence for the occurrence of active starch synthesis and breakdown in leaves of the very low starch *aps1* and *pgm* mutants.

### Additional concluding remarks

**Fig 8:**
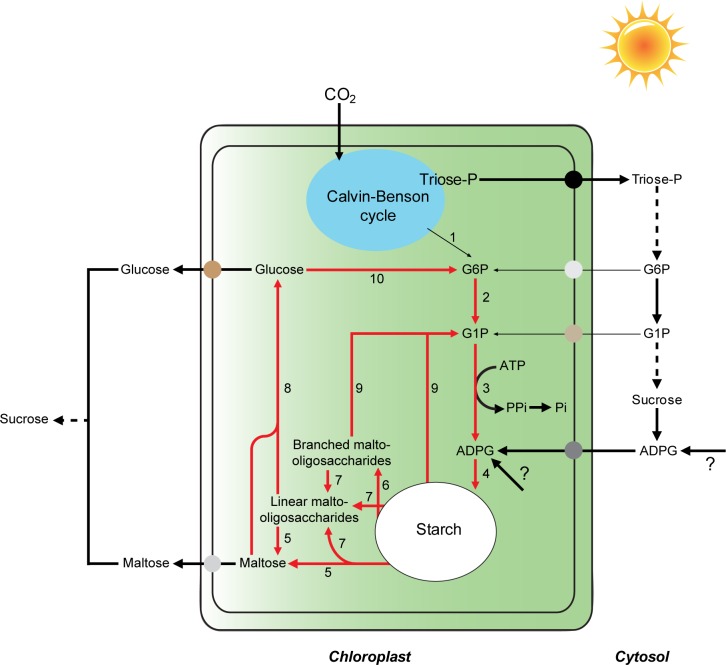
Suggested mechanism of starch metabolism in illuminated leaves of Arabidopsis involving simultaneous synthesis and breakdown of starch. During the day, photosynthetically fixed carbon is either exported to the cytosol as triose phosphates by means of TPT to be subsequently converted into sucrose, and/or retained within the chloroplast to fuel starch biosynthesis. Starch is then degraded to maltose and glucose molecules that are either exported to the cytosol via MEX1 and pGlcT, respectively, or recycled back to starch. This interpretation of leaf starch metabolism previews that (i) hexose-phosphates and/or ADPG occurring in the cytosol enter the chloroplast for subsequent conversion into starch, (ii) CBC and the pPGM-AGP-SS starch biosynthetic pathway are not connected by pPGI, and (iii) pPGM and AGP play important roles not only in the *de novo* synthesis of starch from the CBC, but also in the scavenging of starch breakdown products. The enzyme activities involved are numbered as follows: 1, pPGI; 2, pPGM; 3, AGP; 4, SS; 5, β-amylase; 6, AMY; 7, debranching enzymes; 8, DPE1; 9, SP; 10, hexokinase. Enzymatic reactions involved in starch cycling are indicated with red arrows.

Results presented in this communication provide strong evidence for the occurrence of extensive starch breakdown in Arabidopsis leaves during illumination. The fact that *mex1* and *mex1/pglct* mutants accumulate high levels of maltose in leaves and display a dwarf phenotype under both SD and CL conditions ([40,60,64]; our work, cf. **Fig 2**) strongly indicates that MEX1 and pGlcT not only participate in the export of starch breakdown products during the night but, together with the triose-P translocator (TPT), co-participate in the export of photoassimilates from the chloroplast to the cytosol for their subsequent conversion into sucrose to support growth. Thus, according to this interpretation of starch metabolism, photosynthate export from the chloroplast to the cytosol during illumination would involve the synthesis and subsequent export of starch breakdown products as schematically illustrated in **Fig 8**. This view is consistent with previous reports showing that mesophyll cells of mutants impaired in TPT can export starch breakdown products to the cytosol during illumination [67,68].

The ^14^CO_2_ pulse-chase method employed to investigate the possible occurrence of starch turnover is based on the amylolytic digestion of ethanol precipitates from crude extracts of leaves after ^14^CO_2_ exposure, and measurement of the label in the supernatant. Classic ^14^CO_2_ pulse-chase studies on illuminated leaves showed no significant loss of ^14^C from starch during the chase period [53,54]. Assuming that radioactivity released by amylolytic enzymes is exclusively associated with glucose from starch, these results would indicate that starch degradation does not operate during illumination in leaves [53,54]. However, this interpretation conflicts with the results presented in this work showing that leaves of starch breakdown mutants accumulate higher levels of starch than WT plants when the dark period is omitted (**Fig 1**). Based on the results presented in **Fig 3**, we propose that the persistence of constant radioactivity levels in amylolytic extracts of plants during the chase in ^14^CO_2_ pulse-chase experiments [53,54] could be ascribed, at least partly, to the release by amylolytic enzymes of labeled, non-starch carbon compounds that are not subject to degradation during illumination. Additionally, this phenomenon could be ascribed, at least partly, to the operation of a very efficient mechanism of scavenging of labeled starch breakdown products. In this last respect, we must emphasize that our results of δ^13^C kinetics in WT and *aps1* plants cultured in ^13^CO_2_-enriched environment (**Fig 4**) provide evidence for the occurrence of a mechanism of AGP-mediated starch cycling in illuminated Arabidopsis leaves as schematically illustrated in **Fig 8**. The occurrence of starch cycling is consistent with the interpretation on the starch granule formation according to which debranching enzymes and DPE1 would play important roles not only in starch breakdown, but also in the synthesis and maturation of the starch granule [26,27]. Inherent to this interpretation is that AGP is required for recycling of glucose molecules derived from the DPE1 mediated transformation of maltodextrins.

Labeling kinetics analyses alone do not provide information about the magnitude of metabolic fluxes [50]. Such analyses require data on metabolic pool sizes, partitioning of the pools, enzymatic activities, etc., a task that becomes even more complicated when substrate cycles operate in a given metabolic pathway. Therefore, kinetic analyses of δ^13^C starch conducted in this work do not provide a quantitative estimation of the magnitude of metabolic fluxes involved in synthesis and breakdown of starch, and recycling of breakdown products to starch during illumination. The fact that *aps1/gwd* leaves accumulate 10–15% of the WT starch would indicate that a maximum of 10–15% of the starch accumulated in the leaf is subject to degradation during illumination. This inferred value, however, is an underestimation of the true flux through starch breakdown, since the low starch content of *aps1/gwd* leaves could be partly the consequence of the extremely reduced photosynthetic capacity of the mutant (**S5 Fig**).

Most observations on storage carbohydrate cycling are consistent with a role for the cycle as a switch mechanism, allowing the cell to rapidly utilize storage carbohydrates in adapting to sudden environmental changes. Thus, we hypothesize that starch cycling may entail important advantages such as rapid metabolic channeling toward various pathways, especially in response to physiological and biochemical needs imposed by the environment. Maltose molecules derived from starch breakdown during illumination could play an important role in protecting proteins, membranes and the photosynthetic electron transport in response to temperature stress [69] or in supporting the biosynthesis of proline required to face osmotic stress [46,70]. Hexose-Ps derived from starch breakdown in illuminated leaves could supply carbon to the CBC under photorespiratory conditions [71]. In plants exhibiting high photosynthetic activity and growth, and accumulation of exceptionally high levels of starch in response to signals emitted by microorganisms [43,72,73], G1P molecules derived from starch breakdown could be utilized to produce sulfolipids required for proper functioning of photosynthetic membranes. Furthermore, maltose and glucose derived from starch breakdown could be exported to the cytosol to be converted into hexose-Ps and sucrose.

## Materials and methods

### Plant material and growth conditions

The study was carried out using *Arabidopsis thaliana* WT (ecotypes Col-*0* and Wasilewskija Ws-2) and the *gwd* (SALK_077211), *sex4* (SALK_126784), *bam4* (SALK_037355), *mex1* (SALK_201638), *pglct* (SALK_078684), *bam1/bam3*, *dpe1* (SALK_207318), *amy3/isa3/lda*, *ss4* [58], *aps1* (SALK_040155), *pgm* (GABI_094D07), *aps1/pgm* [39] and pPGI null *pgi1-2* [31,32] mutants. By crossing *mex1* with either *aps1*, *pgm* and *pglct*, self-pollinating the resulting heterozygous mutants, and PCR screening for homozygous progeny using the oligonucleotide primers listed in **S1 Table** we produced the *mex1/aps1*, *mex1/pgm* and *mex1/pglct* double mutants, respectively. Unless otherwise indicated, plants were germinated and permanently cultured on soil in growth chambers under either LD (16 h light (100 μmol photons sec^-1^ m^-2^)/8 h dark photoperiod, 22°C during the light period and 18°C during the dark period) or CL conditions (light intensity of 100 μmol photons sec^-1^ m^-2^, at a constant temperature of 22°C). At the indicated times, leaves were harvested, immediately freeze-clamped, ground to a fine powder in liquid nitrogen with a pestle and mortar, and stored at -80°C.

### ^13^CO_2_ feeding experiments

For ^13^CO_2_ pulse-chase experiments, plants were cultured in growth cabinets under LD conditions (22°C during the light period and 18°C during the dark period). A 50 mL syringe (SGE, Ringwood, Australia) and needle (model microlance 3, BD, Plymount, JK) were filled with 99.9 ‰ ^13^CO_2_ and placed on a syringe pump, which delivered ^13^CO_2_ to the growth cabinet. This system allowed homogenous labeling of the CO_2_ in the growth cabinet throughout the pulse phase. During the chase plants were transferred to a growth cabinet lacking a ^13^CO_2_ enriched atmosphere. At the indicated times, leaves were harvested and stored at -80°C as described above.

### Enzyme assays

One g of the frozen powder (see above) was resuspended at 4°C in 3 mL of 100 mM HEPES (pH 7.5), 2 mM EDTA and 2 mM dithiothreitol, 1 mM PMSF and 10 mL/L protease inhibitor cocktail (Sigma P9599), and centrifuged at 14,000 x g for 20 min. The supernatant was desalted by ultrafiltration on Vivaspin 500 centrifugal concentrator (Sartorius) and the protein extract thus obtained was assayed for enzymatic activities. AGP activity was measured following the two-step assay method described in [57]. SS activity was measured according to [32]. One unit (U) is defined as the amount of enzyme that catalyzes the production of 1 μmol of product per min.

### Western blot analyses

For immunoblot analyses of SS4, protein samples (30 μg protein/lane) were separated on 10% SDS-PAGE, transferred to nitrocellulose filters, and immunodetected by using antisera raised against SS4 as primary antibody [59], and a goat anti-rabbit IgG alkaline phosphatase conjugate (Sigma) as secondary antibody. Non-reducing western blots of the small subunit of AGP (APS1) were conducted as described in [57].

### Iodine staining

Plants harvested at the 18 days after sowing (DAS) stage were fixed by immersion into 3.7% formaldehyde in phosphate buffer. Leaf pigments were then removed in 96% ethanol. Re-hydrated samples were stained in iodine solution (KI 2% (w/v) and I_2_ 1% (w/v)) for 30 min, rinsed briefly in deionized water and photographed.

### Measurement of total C and starch carbon contents in amyloglucosidase digests

A 0.15 g aliquot of the frozen powder of plants (see above) was resuspended in 1 mL of 80% (v/v) ethanol (**S3 Fig**). The ethanol-insoluble material was pelleted by centrifugation, washed three times with 1 mL 80% ethanol, resuspended in 300 μL of 0.2 M KOH and heated for 1 h at 95°C as described in [54,74]. The insoluble material was pelleted by centrifugation, ca. 15 μL of 1 M HCl was added to the supernatant until pH was adjusted to ca. 5.0, and phosphate buffer (pH 4.6) was added. Then, starch was digested to glucose by the addition of 0.5 U of amyloglucosidase from *Aspergillus niger* (Sigma Aldrich GmbH, Munich, Germany. Ref. A1602). Control samples contained heat-denatured amyloglucosidase. The digests and controls were adjusted to 75% (v/v) methanol and 1% (w/v) KCl, and incubated for 1 h at 4°C. After centrifugation at 15,000 x g for 20 min, the methanol/KCl-insoluble material was removed. The supernatant was dried at 60°C in a drying oven, and water (100 μL) was added (**S3 Fig**). The resulting sample (designated as “amyloglucosidase digest”) was used for subsequent measurements of TOC and starch contents. For measurement of TOC content, aliquots of the amyloglucosidase digests and controls were molecularly filtered through thoroughly rinsed VIVASPIN (10,000 MWCO) membranes, loaded in tin containers (5 x 9 mm) and dried in a drying oven at 50°C for 15 hours. TOC content in the residue was then determined using a NC 2500 elemental analyzer (Carlo Erba Instrumentazione, Milan, Italy) as indicated in http://www.ru.nl/science/gi/facilities/elemental-analysis/cn-elemental/. Starch carbon content was calculated considering that 1 mol of glucose contains 72 g of carbon.

### HPLC/IRMS analyses of δ^13^C in starch in leaves of ^13^CO_2_ pulse-chased plants

HPLC/IRMS analyses of δ^13^C in the glucose molecules of amyloglucosidase digests (**S3 Fig**) obtained from leaves of ^13^CO_2_ pulse-chased plants and controls were conducted essentially as described in [61]. High-performance anion-exchange chromatography was carried out on a Thermo Surveyor system consisting of an HPLC pump (MS Pump Plus) and autoinjector (Autosampler Plus; Thermo Electron, Bremen, Germany), fitted with a CarboPac PA20 guard and narrow-bore analytical column (3 x 150 mm; Dionex Benelux, Amsterdam, The Netherlands) and eluted at 300 μL/min isocratically with 1 mM NaOH. The column was regularly regenerated with 200 mM NaOH. The HPLC system was coupled to the IRMS instrument by an LC Isolink interface (Thermo Electron). The temperature of the oxidation reactor was set at 99.9°C. The flow rates of the acid (1.5 M H_3_PO_4_) and oxidant reagents (0.3 M Na_2_S_2_O_8_) were each 50 μL/min.

Isotopic ratio measurements were carried out on a Delta V Advantage isotope ratio mass spectrometer (Thermo Electron). The reference gas was regularly calibrated against phthalic acid (Schimmelmann, Bloomington, IN, USA) with a δ^13^C value of -27.21 ± 0.02%. Stable carbon isotope ratios were reported in the delta-notation:

$${\text{d}}^{13}\text{C}\;\left(‰\right)=\left({\text{R}}_{\text{sample}}{/\text{R}}_{\text{VPDB}}\right)-1)\;x\;1000$$

where R_sample_ and R_VPDB_ are the ^13^C/^12^C ratio in the sample and international standard (Vienna Pee Dee Belemnite), respectively. Peak identification of glucose was based on retention times in comparison with external standards. Glucose concentration measurements were based on peak areas of the separated compounds and calibrated against external standards.

### HPLC/IRMS analyses of δ^13^C in soluble sugars in leaves of ^13^CO_2_ pulse-chased plants

A 0.15 g aliquot of the frozen powder of leaves of ^13^CO_2_ pulse-chased plants (see above) was resuspended in 1 mL of 80% (v/v) ethanol (**S3 Fig**). After centrifugation at 15,000 x g for 20 min, the supernatant was dried at 60°C in a drying oven, and water (100 μL) was added (**S3 Fig**). Sucrose, glucose and fructose molecules were then subject to HPLC/IRMS analyses of δ^13^C.

### Analytical procedures

For measurement of maltose and maltotriose contents, 0.15 g of the frozen powder (see above) was resuspended in 1 mL of 80% ethanol, left at 70°C for 90 min and centrifuged at 13,000 x g for 10 min (**S2 Fig**). Maltose and maltotriose from supernatants were then determined by HPAEC-PAD on a DX-500 Dionex system by gradient separation with a CarboPac PA20 column according to the application method suggested by the supplier. We checked the reliability of the method of maltose detection and measurement by adding known amounts of maltose to supernatants previously digested with maltase. Maltose was also measured by GC-MS essentially as described in [75]. The GC-MS system consisted of a 7890A GC device coupled to a 5975C Inert XL MSD mass selective detector (Agilent Technologies, Santa Clara, USA). A volume of 1 μL was injected on an Agilent J&W HP-5ms column (diameter, 0.25 mm; film thickness, 0.25 μm; length, 30 m) with a 0.6 mL/min helium flow. The injection parameters were as follows: splitless injection at 230°C with a 1-min purge at 20 mL/min. The temperature gradient was 1 min at 70°C; 9°C/min until 320°C; and then 10 min at 320°C, and the solvent delay was 5.4 min. The source was set to 250°C and 70 eV, scanning at 20 scans/min, from 70 to 600 m/z Acquisition was performed with Chemstation software (Agilent).

Starch content was determined by measuring the glucose content in the amyloglucosidase digests both spectrophotometrically (using an amyloglucosydase–based test kit, r-biopharm, ref. 10207748035) and by HPAEC-PAD. Chlorophyll content was quantified according to [76].

### Gas exchange determinations

Gas exchange determinations were conducted as described in [73] using a LI-COR 6400 gas exchange portable photosynthesis system (LI-COR, Lincoln, Nebraska, USA).

### Statistical analysis

Presented data are the means (± SE) of three independent experiments, with 3 replicates for each experiment. The significance of differences was statistically evaluated with Student´s t-test using the SPSS software. Differences were considered significant if p<0.05.

## Supporting information

S1 Fig Metabolic schemes of diurnal starch synthesis and nocturnal starch degradation. (A) During the day, photosynthetically fixed carbon is either retained within the chloroplast to fuel the synthesis of transitory starch, or exported to the cytosol as triose phosphates by means of TPT to be subsequently converted into sucrose. (B) During the night, starch is remobilized thereby providing maltose and glucose molecules that are exported to the cytosol and metabolized to support sucrose synthesis and growth. The enzyme activities involved are numbered as follows: 1, pPGI; 2, pPGM; 3, AGP; 4, SS; 5, β-amylase; 6, AMY; 7, debranching enzymes; 8, DPE1; 9, SP. In “B”, maltose and glucose are transported from plastid to the cytosol via the MEX1 and pGlcT transporters, respectively.
(EPS)

S2 Fig Biochemical characterization of WT (Col-*0*) plants and the indicated starch breakdown mutants. (A) Net photosynthetic CO_2_ fixation rates, (B) total AGP and SS activities, (C) non-reducing western blot of AGP and (D) western blot of SS4. In “A”, gas exchange determinations were conducted at 25°C with a photosynthetic photon flux density of 350 μmol m^-2^ s^-1^ and with a CO_2_ concentration of 450 μmol mol^-1^.
(EPS)

S3 Fig Preparation of samples for measurement of TOC and starch contents, and HPLC/IRMS analyses of starch. (EPS)

S4 Fig **Time-course of starch content in leaves of (A) Col-*0* and *aps1* plants, and (B) Ws-2 and *pgi1-2* plants cultured under LD conditions.** In “A” and “B”, the experiment was conducted using plants at the 26 DAS and 22 DAS growth stages, respectively. Values represent the means ± SE determined from three independent experiments using 6 plants in each experiment.
(EPS)

S5 Fig Incorporation of the *gwd* mutation exerts a negative effect on photosynthesis in CL grown *aps1* plants. The graphic represents the curves of net CO_2_ assimilation rate (*A*_*n*_) versus intercellular CO_2_ concentration (*Ci*) in 20 DAS WT (Col-*0*), *aps1*, *gwd* and *aps1/gwd* leaves.
(EPS)

S1 Table Primers used for PCR screening of mutants. (DOCX)

## References

[1] Newsholme EA, Challiss RAJ, Crabtree B. Substrate cycles: their role in improving sensitivity in metabolic control. Trends Biochem Sci. 1984;9: 277–280.

[2] Portais JC, Delort AM. Carbohydrate cycling in micro-organisms: What can ^13^C-NMR tell us? FEMS Microbiology Reviews. 2002;26: 375–402. 12413666 10.1111/j.1574-6976.2002.tb00621.x

[3] Baranyai JM, Blum JJ. Quantitative analysis of intermediary metabolism in rat hepatocytes incubated in the presence and absence of ethanol with a substrate mixture including ketoleucine. Biochem J. 1989;258: 121–140 2930501 10.1042/bj2580121 PMC1138332

[4] Hill SA, ap Rees T. Fluxes of carbohydrate metabolism in ripening bananas. Planta. 1994;192: 52–60.

[5] Dieuaide-Noubhani M, Raffard G, Canioni P, Pradet A, Raymond P. Quantification of compartmented metabolic fluxes in maize root tips using isotope distribution from ^13^C- or ^14^C-labeled glucose. J Biol Chem. 1995;270: 13147–13159. 7768910 10.1074/jbc.270.22.13147

[6] Alonso AP, Vigeolas H, Raymond P, Rolin D, Dieuaide-Noubhani M. A new substrate cycle in plants. Evidence for a high glucose-phosphate-to-glucose turnover from in vivo steady-state and pulse-labelling experiments with [^13^C] glucose and [^14^C] glucose. Plant Physiol. 2005;138: 2220–2232. 10.1104/pp.105.062083 16024683 PMC1183409

[7] Wendler R, Veith R, Dancer J, Stitt M, Komor E. Sucrose storage in cell suspension cultures of *Saccharum* sp. (sugarcane) is regulated by a cycle of synthesis and degradation. Planta. 1991;183: 31–39. 10.1007/BF00197564 24193530

[8] Alonso AP, Raymond P, Rolin D, Dieuaide-Noubhani M. Substrate cycles in the central metabolism of maize root tips under hypoxia. Phytochemistry. 2007;68: 2222–2231. 10.1016/j.phytochem.2007.04.022 17559894

[9] Navas MA, Cerdán S, Gancedo JM. Futile cycles in *Saccharomyces cerevisiae* strains expressing the gluconeogenic enzymes during growth on glucose. Proc Natl Acad Sci U S A. 1993;90: 1290–1294. 8381962 10.1073/pnas.90.4.1290 PMC45858

[10] Jones ME, Berry MN, Phillips JW. Futile cycles revisited: A Markov chain model of simultaneous glycolysis and gluconeogenesis. J Theor Biol. 2002;217: 509–523. 12234757 10.1006/jtbi.2002.3042

[11] Parrou JL, Teste MA, François J. Effects of various types of stress on the metabolism of reserve carbohydrates in *Saccharomyces cerevisiae*: Genetic evidence for a stress-induced recycling of glycogen and trehalose. Microbiology. 1997;143: 1891–1900. 10.1099/00221287-143-6-1891 9202465

[12] Bago B, Pfeffer PE, Douds DD Jr., Brouillette J, Bécard G, Shachar-Hill Y. Carbon metabolism in spores of the arbuscular mycorrhizal fungus *Glomus intraradices* as revealed by nuclear magnetic resonance spectroscopy. Plant Physiol. 1999;121: 263–272. 10482682 10.1104/pp.121.1.263 PMC59376

[13] Geigenberger P, Stitt M. A futile cycle of sucrose synthesis and degradation is involved in regulating partitioning between sucrose, starch and respiration in cotyledons of germinating *Ricinus communis* L. seedlings when phloem transport is inhibited. Planta. 1991;185: 81–90. 10.1007/BF00194518 24186283

[14] Nguyen-Quoc B, Foyer CH. A role for “futile cycles” involving invertase and sucrose synthase in sucrose metabolism of tomato fruit. J Exp Bot. 2001;52: 881–889. 11432905 10.1093/jexbot/52.358.881

[15] David M, Petit WA, Laughlin MR, Shulman RG, King JE, Barrett EJ. Simultaneous synthesis and degradation of rat liver glycogen. An in vivo nuclear magnetic resonance spectroscopic study. J Clin Invest. 1990;86: 612–617. 10.1172/JCI114752 2117024 PMC296768

[16] Massillon D, Bollen M, De Wulf H, Overloop K, Vanstapel F, Van Hecke P, et al Demonstration of a glycogen/glucose 1-phosphate cycle in hepatocytes from fasted rats. Selective inactivation of phosphorylase by 2-deoxy-2-fluoro-alpha-D-glucopyranosyl fluoride. Journal of Biological Chemistry. 1995;270: 19351–19356. 7642613 10.1074/jbc.270.33.19351

[17] Bollen M, Keppens S, Stalmans W. Specific features of glycogen metabolism in the liver. Biochem J. 1998;336: 19–31. 9806880 10.1042/bj3360019 PMC1219837

[18] Lehmann M, Wöber G. Accumulation, mobilization and turn-over of glycogen in the blue-green bacterium *Anacystis nidulans*. Arch Microbiol. 1976;111: 93–97. 828031 10.1007/BF00446554

[19] Gaudet G, Forano E, Dauphin G, Delort AM. Futile cycling of glycogen in *Fibrobacter succinogenes* as shown by in situ ^1^H-NMR and ^13^C-NMR investigation. Eur J Biochem. 1992;207: 155–162. 1628646 10.1111/j.1432-1033.1992.tb17032.x

[20] Matheron C, Delort AM, Gaudet G, Forano E, Liptaj T. ^13^C and ^1^H nuclear magnetic resonance study of glycogen futile cycling in strains of the genus *Fibrobacter*. Appl Environ Microbiol. 1998;64: 74–81. 12033219 10.1128/aem.64.1.74-81.1998 PMC124674

[21] Belanger AE, Hatfull GF. Exponential-phase glycogen recycling is essential for growth of *Mycobacterium smegmatis*. J Bacteriol. 1999;181: 6670–6678. 10542168 10.1128/jb.181.21.6670-6678.1999 PMC94131

[22] Guedon E, Desvaux M PH. Kinetic analysis of *Clostridium cellulolyticum* carbohydrate metabolism: importance of glucose 1-phosphate and glucose 6-phosphate branch points for distribution of carbon fluxes inside and outside cells as revealed by steady-state continuous culture. J Bacteriol. 2000;182: 2010–2017. 10715010 10.1128/jb.182.7.2010-2017.2000 PMC101914

[23] Montero M, Almagro G, Eydallin G, Viale AM, Muñoz FJ, Bahaji A, et al *Escherichia coli* glycogen genes are organized in a single *glgBXCAP* transcriptional unit possessing an alternative suboperonic promoter within *glgC* that directs *glgAP* expression. Biochem J. 2011;433: 107–117. 10.1042/BJ20101186 21029047

[24] Almagro G, Viale AM, Montero M, Rahimpour M, Muñoz FJ, Baroja-Fernández E, et al Comparative genomic and phylogenetic analyses of gammaproteobacterial glg genes traced the origin of the *Escherichia coli* glycogen *glgBXCAP* operon to the last common ancestor of the sister orders *Enterobacteriales* and *Pasteurellales*. PLoS One. Public Library of Science; 2015;10: e0115516 10.1371/journal.pone.0115516 25607991 PMC4301808

[25] Rao IM, Terry N. Leaf phosphate status, photosynthesis, and carbon partitioning in sugar beet (IV. Changes with time following increased supply of phosphate to low-phosphate plants). Plant Physiol. 1995;107: 1313–1321. 12228438 10.1104/pp.107.4.1313 PMC157266

[26] Myers A.M., Morell M.K., James M.G., Ball SG. Recent progress towards understanding the biogenesis of the amylopectin crystal. Plant Physiol. 2000;122: 989–997. 10759494 10.1104/pp.122.4.989 PMC1539245

[27] Ball SG, Morell MK. From bacterial glycogen to starch: Understanding the biogenesis of the plant starch granule. Annu Rev Plant Biol. 2003;54: 207–233. 10.1146/annurev.arplant.54.031902.134927 14502990

[28] Streb S, Zeeman SC. Starch Metabolism in Arabidopsis. Arab B. 2012;10: e0160. 10.1199/tab.0160 PMC3527087 23393426

[29] Kleczkowski LA. A phosphoglycerate to inorganic phosphate ratio is the major factor in controlling starch levels in chloroplasts via ADP-glucose pyrophosphorylase regulation. FEBS Lett. 1999;448: 153–156. 10217430 10.1016/s0014-5793(99)00346-4

[30] Kleczkowski LA. Is leaf ADP-glucose pyrophosphorylase an allosteric enzyme? Biochim Biophys Acta. 2000;1476: 103–108. 10606772 10.1016/s0167-4838(99)00229-0

[31] Kunz HH, Häusler RE, Fettke J, Herbst K, Niewiadomski P, Gierth M, et al The role of plastidial glucose-6-phosphate/phosphate translocators in vegetative tissues of *Arabidopsis thaliana* mutants impaired in starch biosynthesis. Plant Biol. 2010;12: 115–128. 10.1111/j.1438-8677.2010.00349.x 20712627

[32] Bahaji A, Sánchez-López ÁM, De Diego N, Muñoz FJ, Baroja-Fernández E, Li J, et al Plastidic phosphoglucose isomerase is an important determinant of starch accumulation in mesophyll cells, growth, photosynthetic capacity, and biosynthesis of plastidic cytokinins in Arabidopsis. PLoS One. 2015;10: e0119641 10.1371/journal.pone.0119641 25811607 PMC4374969

[33] Caspar T, Huber SC, Somerville C. Alterations in growth, photosynthesis, and respiration in a starchless mutant of *Arabidopsis thaliana* (L.) deficient in chloroplast phosphoglucomutase activity. Plant Physiol. 1985;79: 11–17. 16664354 10.1104/pp.79.1.11 PMC1074821

[34] Kofler H, Häusler RE, Schulz B, Gröner F, Flügge UI, Weber a. Molecular characterisation of a new mutant allele of the plastid phosphoglucomutase in Arabidopsis, and complementation of the mutant with the wild-type cDNA. Mol Gen Genet. 2000;263: 978–986. 10954083 10.1007/pl00008698

[35] Lin TP, Caspar T, Somerville C, Preiss J. Isolation and characterization of a starchless mutant of *Arabidopsis thaliana* (L.) Heynh lacking ADPglucose pyrophosphorylase activity. Plant Physiol. 1988;86: 1131–1135. 16666044 10.1104/pp.86.4.1131 PMC1054640

[36] Wang S, Lue W, Yu T, Long J, Wang C, Eimert K, et al Characterization of ADG1, an Arabidopsis locus encoding for ADPG pyrophosphorylase small subunit, demonstrates that the presence of the small subunit is required for large subunit stability. Plant J. Blackwell Science, Ltd; 1998;13: 63–70. 9680965 10.1046/j.1365-313x.1998.00009.x

[37] Ventriglia T, Kuhn ML, Ruiz MT, Ribeiro-Pedro M, Valverde F, Ballicora M a, et al Two Arabidopsis ADP-glucose pyrophosphorylase large subunits (APL1 and APL2) are catalytic. Plant Physiol. 2008;148: 65–76. 10.1104/pp.108.122846 18614708 PMC2528121

[38] Bahaji A, Li J, Ovecka M, Ezquer I, Muñoz FJ, Baroja-Fernández E, et al *Arabidopsis thaliana* mutants lacking ADP-glucose pyrophosphorylase accumulate starch and wild-type ADP-Glucose content: Further evidence for the occurrence of important sources, other than ADP-glucose pyrophosphorylase, of ADP-glucose linked to leaf starch biosynthesis. Plant Cell Physiol. 2011;52: 1162–1176. 10.1093/pcp/pcr067 21624897

[39] Bahaji A, Baroja-Fernández E, Sánchez-López ÁM, Muñoz FJ, Li J, Almagro G, et al HPLC-MS/MS analyses show that the near-starchless *aps1* and *pgm* leaves accumulate wild type levels of ADPglucose: further evidence for the occurrence of important ADPglucose biosynthetic pathway(s) alternative to the pPGI-pPGM-AGP pathway. PLoS One. 2014;9: e104997 10.1371/journal.pone.0104997 25133777 PMC4136846

[40] Cho MH, Lim H, Shin DH, Jeon JS, Bhoo SH, Park Y Il, et al Role of the plastidic glucose translocator in the export of starch degradation products from the chloroplasts in *Arabidopsis thaliana*. New Phytol. 2011;190: 101–112. 10.1111/j.1469-8137.2010.03580.x 21175634

[41] Malinova I, Mahlow S, Alseekh S, Orawetz T, Fernie AR, Baumann O, et al Double knockout mutants of Arabidopsis grown under normal conditions reveal that the plastidial phosphorylase isozyme participates in transitory starch metabolism. Plant Physiol. 2014;164: 907–921. 10.1104/pp.113.227843 24302650 PMC3912115

[42] Bahaji A, Li J, Sánchez-López ÁM, Baroja-Fernández E, Muñoz FJ, Ovecka M, et al Starch biosynthesis, its regulation and biotechnological approaches to improve crop yields. Biotechnology Advances. 2014;32: 87–106. 10.1016/j.biotechadv.2013.06.006 23827783

[43] Sánchez-López ÁM, Bahaji A, De Diego N, Baslam M, Li J, Muñoz FJ, Almagro G, et al Arabidopsis responds to *Alternaria alternata* volatiles by triggering plastidic phosphoglucose isomerase-independent mechanisms. Plant Physiol. 2016;172: 1989–2001 10.1104/pp.16.00945 27663407 PMC5100789

[44] Mikkelsen R, Mutenda KE, Mant A, Schürmann P, Blennow A. Alpha-glucan, water dikinase (GWD): a plastidic enzyme with redox-regulated and coordinated catalytic activity and binding affinity. Proc Natl Acad Sci U S A. 2005;102: 1785–1790. 10.1073/pnas.0406674102 15665090 PMC547843

[45] Sparla F, Costa A, Lo Schiavo F, Pupillo P, Trost P. Redox regulation of a novel plastid-targeted beta-amylase of Arabidopsis. Plant Physiol. 2006;141: 840–850. 10.1104/pp.106.079186 16698902 PMC1489908

[46] Valerio C, Costa A, Marri L, Issakidis-Bourguet E, Pupillo P, Trost P, et al Thioredoxin-regulated beta-amylase (BAM1) triggers diurnal starch degradation in guard cells, and in mesophyll cells under osmotic stress. J Exp Bot. 2011;62: 545–555. 10.1093/jxb/erq288 20876336 PMC3003804

[47] Silver DM, Silva LP, Issakidis-Bourguet E, Glaring MA, Schriemer DC, Moorhead GBG. Insight into the redox regulation of the phosphoglucan phosphatase SEX4 involved in starch degradation. FEBS Journal. 2012;280: 538–548. 10.1111/j.1742-4658.2012.08546.x 22372537

[48] Santelia D, Trost P, Sparla F. New insights into redox control of starch degradation. Current Opinion in Plant Biology. 2015;25: 1–9. 10.1016/j.pbi.2015.04.003 25899330

[49] Caspar T, Lin TP, Kakefuda G, Benbow L, Preiss J, Somerville C. Mutants of Arabidopsis with altered regulation of starch degradation. Plant Physiol. 1991;95: 1181–1188. 16668109 10.1104/pp.95.4.1181 PMC1077670

[50] Szecowka M, Heise R, Tohge T, Nunes-Nesi A, Vosloh D, Huege J, et al Metabolic fluxes in an illuminated Arabidopsis rosette. Plant Cell. 2013;25: 694–714. 10.1105/tpc.112.106989 23444331 PMC3608787

[51] Stitt M, Heldt HW. Simultaneous synthesis and degradation of starch in spinach chloroplasts in the light. Biochim Biophys Acta- Bioenerg. 1981;638: 1–11.

[52] Lozovaya V V., Zabotina O a., Widholm JM. Synthesis and turnover of cell-wall polysaccharides and starch in photosynthetic soybean suspension cultures. Plant Physiol. 1996;111: 921–929. 12226338 10.1104/pp.111.3.921 PMC157911

[53] Zeeman SC, Tiessen A, Pilling E, Kato KL, Donald AM, Smith AM. Starch synthesis in Arabidopsis. Granule synthesis, composition, and structure. Plant Physiol. 2002;129: 516–529. 10.1104/pp.003756 12068097 PMC161669

[54] Li B, Geiger DR, Shieh W-J. Evidence for circadian regulation of starch and sucrose synthesis in sugar beet leaves. Plant Physiol. 1992;99: 1393–1399 16669050 10.1104/pp.99.4.1393 PMC1080638

[55] Zhang X, Myers AM, James MG. Mutations affecting starch synthase III in Arabidopsis alter leaf starch structure and increase the rate of starch synthesis. Plant Physiol. 2005;138: 663–674. 10.1104/pp.105.060319 15908598 PMC1150387

[56] Richter A, Wanek W. Preparation of starch and soluble sugars of plant material for the analysis of carbon isotope composition: a comparison of methods. Rapid Commun Mass Spectrom. 2009;23: 2476–2488. 10.1002/rcm.4088 19603463

[57] Li J, Almagro G, Muñoz FJ, Baroja-Fernández E, Bahaji A, Montero M, et al Post-translational redox modification of ADP-glucose pyrophosphorylase in response to light is not a major determinant of fine regulation of transitory starch accumulation in arabidopsis leaves. Plant Cell Physiol. 2012;53: 433–444. 10.1093/pcp/pcr193 22210900

[58] Szydlowski N, Ragel P, Raynaud S, Lucas MM, Roldán I, Montero M, et al Starch granule initiation in Arabidopsis requires the presence of either class IV or class III starch synthases. Plant Cell. 2009;21: 2443–2457. 10.1105/tpc.109.066522 19666739 PMC2751949

[59] Gámez-Arjona FM, Li J, Raynaud S, Baroja-Fernández E, Muñoz FJ, Ovecka M, et al Enhancing the expression of starch synthase class IV results in increased levels of both transitory and long-term storage starch. Plant Biotechnol J. 2011;9: 1049–1060. 10.1111/j.1467-7652.2011.00626.x 21645200

[60] Stettler M, Eicke S, Mettler T, Messerli G, Hörtensteiner S, Zeeman SC. Blocking the metabolism of starch breakdown products in Arabidopsis leaves triggers chloroplast degradation. Mol Plant. 2009;2: 1233–1246. 10.1093/mp/ssp093 19946617 PMC2782796

[61] Boschker HTS, Moerdijk-Poortvliet TCW, van Breugel P, Houtekamer M Middelburg JJ. A versatile method for stable carbon isotope analysis of carbohydrates by high-performance liquid chromatography/isotope ratio mass spectrometry. Rapid Commun Mass Spectrom. 2008;22: 3902–3908. 10.1002/rcm.3804 18980267

[62] Moerdijk-Poortvliet TCW, Schierbeek H, Houtekamer M, Van Engeland T, Derrien D, Stal LJ, et al Comparison of gas chromatography/isotope ratio mass spectrometry and liquid chromatography/isotope ratio mass spectrometry for carbon stable-isotope analysis of carbohydrates. Rapid Commun Mass Spectrom. 2015;29: 1205–1214. 10.1002/rcm.7217 26395604

[63] Weise SE, Weber APM, Sharkey TD. Maltose is the major form of carbon exported from the chloroplast at night. Planta. 2004;218: 474–482. 10.1007/s00425-003-1128-y 14566561

[64] Niittylä T, Messerli G, Trevisan M, Chen J, Smith AM, Zeeman SC. A previously unknown maltose transporter essential for starch degradation in leaves. Science. 2004;303: 87–89. 10.1126/science.1091811 14704427

[65] Sun J, Okita TW, Edwards GE. Modification of carbon partitioning, photosynthetic capacity, and O2 sensitivity in Arabidopsis plants with low ADP-glucose pyrophosphorylase activity. Plant Physiol. 1999;119: 267–276. 9880369 10.1104/pp.119.1.267 PMC32229

[66] Ragel P, Streb S, Feil R, Sahrawy M, Annunziata MG, Lunn JE, et al Loss of starch granule initiation has a deleterious effect on the growth of Arabidopsis plants due to an accumulation of ADP-glucose. Plant Physiol. 2013;163: 75–85. 10.1104/pp.113.223420 23872660 PMC3762666

[67] Häusler RE, Schlieben NH, Schulz B, Flügge U-I. Compensation of decreased triose phosphate/phosphate translocator activity by accelerated starch turnover and glucose transport in transgenic tobacco. Planta. 1998;204: 366–376. 10.1007/s004250050268 9530880

[68] Schneider A, Hausler RE, Kolukisaoglu U, Kunze R, van der Graaff E, Schwacke R, et al An *Arabidopsis thaliana* knock-out mutant of the chloroplast triose phosphate/phosphate translocator is severely compromised only when starch synthesis, but not starch mobilisation is abolished. Plant J. Blackwell Science Ltd; 2002;32: 685–699. 12472685 10.1046/j.1365-313x.2002.01460.x

[69] Kaplan F, Guy CL. β-amylase induction and the protective role of maltose during temperature shock. Plant Physiol. 2004;135: 1674–1684. 10.1104/pp.104.040808 15247404 PMC519081

[70] Zanella M, Borghi GL, Pirone C, Thalmann M, Pazmino D, Costa A, et al β-amylase 1 (BAM1) degrades transitory starch to sustain proline biosynthesis during drought stress. J Exp Bot. 2016;67: 1819–1826. 10.1093/jxb/erv572 26792489

[71] Weise SE, Schrader SM, Kleinbeck KR, Sharkey TD. Carbon balance and circadian regulation of hydrolytic and phosphorolytic breakdown of transitory starch. Plant Physiol. 2006;141: 879–886. 10.1104/pp.106.081174 16698896 PMC1489887

[72] Zhang H, Xie X, Kim MS, Kornyeyev DA, Holaday S, Paré PW. Soil bacteria augment Arabidopsis photosynthesis by decreasing glucose sensing and abscisic acid levels in planta. Plant J. 2008;56: 264–273. 10.1111/j.1365-313X.2008.03593.x 18573192

[73] Sánchez-López ÁM, Baslam M, De Diego N, Muñoz FJ, Bahaji A, Almagro G, et al Volatile compounds emitted by diverse phytopathogenic microorganisms promote plant growth and flowering through cytokinin action. Plant Cell Environ. 2016. 10.1111/pce.12759 27092473

[74] Fondy BR, Geiger DR. Diurnal changes in allocation of newly fixed carbon in exporting sugar beet leaves. Plant Physiol. 1985; 78: 753–757 16664319 10.1104/pp.78.4.753 PMC1064816

[75] Bruggeman Q, Prunier F, Mazubert C, Bont L de, Garmier M, Lugan R, et al Involvement of Arabidopsis hexokinase 1 in cell death mediated by myo-inositol accumulation. Plant Cell. 2015;27: 1801–1814. 10.1105/tpc.15.00068 26048869 PMC4498202

[76] Lichtenthaler HK. Chlorophylls and carotenoids: Pigments of photosynthetic biomembranes. Methods Enzymol. 1987;148: 350–382.

