# Supplementary material for: Genetic and isotope ratio mass spectrometric evidence for the occurrence of starch degradation and cycling in illuminated Arabidopsis leaves
Source: PLoS One. 2017 Feb 2;12(2):e0171245. doi: 10.1371/journal.pone.0171245 (PMC5289593; doi:10.1371/journal.pone.0171245)
Supplement: S1 Table — (DOCX) [file pone.0171245.s006.docx]

**S1 Table:** Primers used for PCR screening of mutants.

| **Mutant** | **Primer** | **Sequence** |
| --- | --- | --- |
| *aps1* (SALK_040155) | RP (*aps1*) | 5’-acacacagccgcgttatttaccaccg-3’ |
|  | LP (*aps1*) | 5’-gttttaacatctttaaaacgaataatg-3’ |
|  | LBb1 (T-DNA) | 5’-gcgtggaccgcttgctgcaact-3’ |
|  |  |  |
| *mex1* (SALK_201638) | RP (*mex1*) | 5’-aaacccggacaacataaaagg-3’ |
|  | LP (*mex1*) | 5’-tgtgatttatctgggccaatc-3’ |
|  | LBb1 (T-DNA) | 5’-gcgtggaccgcttgctgcaact-3’ |
|  |  |  |
| *pglct* (SALK_078684) | RP (*pglct*) | 5’-ttcgggatgcaaatatctctg-3’ |
|  | LP (*pglct*) | 5’-gatagctggattaccccttgc-3’ |
|  | LBb1 (T-DNA) | 5’-gcgtggaccgcttgctgcaact-3’ |
|  |  |  |
| *pgm* (GABI_094D07) | RP (*pgm*) | 5’-aatacataccggtttggctcc-3’ |
|  | LP (*pgm*) | 5’-ttgggattctccactttgttg-3’ |
|  | GABI (T-DNA) | 5’-cccatttggacgtgaatgtagacac-3’ |
